# Supplementary material for: Inoculation reduces social media engagement with affectively polarized content in the UK and US
Source: Commun Psychol. 2025 Jan 26;3:11. doi: 10.1038/s44271-025-00189-7 (PMC11769841; doi:10.1038/s44271-025-00189-7)
Supplement: Supplementary file 2 — Supplementary Information [file 44271_2025_189_MOESM2_ESM.pdf]

Supplementary Information for

Inoculation reduces social media engagement with affectively polarized content

Fintan Smith, Almog Simchon, Dawn Holford and Stephan Lewandowsky

almogsi@post.bgu.ac.il

This PDF file includes:

Supplementary text
Fig. S1
Tables S1 to S20
SI References

Supporting Information Text

Supplementary Notes.

Analysis. All transformations and analyses were carried out in R. R code and data can be found at https://zenodo.org/record/8343806 and https://zenodo.org/record/8343729 respectively. Analysis of variance (ANOVA) was conducted using the 'afex' package for R (1). Mediation analysis were conducted using the 'stats' and 'mediation' packages for R (2, 3) and assumption testing was carried out using the 'car' (4) and 'performance' (5) packages for R.

Stimuli. To ensure that derogating synthetic headlines were rated as being more derogating than non-derogating headlines, a validation study was performed. Ethical approval for the validation study was gained from the University of Bristol (application number: 1447480). We recruited participants via a YouGov omnibus survey, that employed quota sampling to garner a nationally and politically representative sample of 1,600 adults in Britain. In total, 1,565 respondents consented to take part.

Each participant saw one of the twelve synthetic headlines and was asked to rate the extent to which headlines were derogatory to any particular group of people. We ran a 2 x 2 x 2 ANOVA with past vote in the EU referendum (as stored by YouGov), the side with which the article was congruent (i.e. either Leave or Remain) and whether the article was derogatory or not as fixed factors. Participants who had not voted in the 2016 EU referendum were excluded, meaning our final $N =$ 1245. This confirmed that derogatory stimuli were perceived to be significantly more derogatory than non-derogatory stimuli ($F(1, 1237) = 176.36$, $p < .001$, $\eta^2_G = .13$) (see Table S5 and Figure S1).

Mediation Analysis. We predicted that the effect of our inoculation upon sharing of affectively polarised content would be mediated by affective polarisation. To test this hypothesis, mediation analysis was performed using the 'mediation' package in R. (3). Incomplete cases (n=118) were removed from the analysis, meaning N=642. Whilst the effectiveness of the inoculation in reducing affective polarisation score ($b = 10.98$, $t(641) = -5.18$, $p < .001$) and likelihood to share affectively polarised content was confirmed ($b = .19$, $t(641) = -2.86$, $p < .01$), the mediator (polarisation score) did not retain significance when the predictor (video condition) was added to the model ($b = .0007$, $t(640) = -.047$, $p = .639$), suggesting that affective polarization score did not significantly mediate the effect of the video upon sharing as we had predicted(6).

Analysis of emoji reactions. Seven binomial logistic regression models were constructed using the 'stats' package for R (2). The first model tested the effect of both video condition and stimuli type on the likelihood of reacting with any of the emojis to the stimuli. A separate model was then constructed to assess the effect of video condition and stimuli type on the likelihood of selecting any of the emoji reactions (see Table S6).

Engagement analyses. Full ANOVA tables for pre-registered analyses of individual engagement measures of sharing are presented in S7. The pre-registed ANOVA presented in S9 confirms that there was no significant interaction between the derogating technique employed in stimuli and the effect of the inoculation.

Validation of Brexit identity strength. Based on a measure employed by previous studies on polarization conducted (e.g. 7). The measure consists of five sub-components measured on five-point Likert agree-disagree scales. Higher average scores indicate stronger 'Remainer' or 'Leaver' identity.

To test the suitability of a one factor solution, a confirmatory factor analysis was performed using the 'lavaan' package for R (Rosseel, 2012). The data was deemed to be suitable for factor analysis: KMO = .84, suggesting sampling was adequate, and Bartlett's test was significant p < .001, suggesting normality (8, 9). Chi-square was significant, χ^2(N = 695, df = 5), = 20.2, $p = .001$, suggesting poor fit, however Chi-square is highly sensitive to sample size and so other measures of fit were consulted (10).

Dynamic cut-offs for measures of fit were calculated at 95% confidence level using the 'dynamic fit shiny app' for R (11). Observed values satisfied all other measures of fit (see Tables S11 for dynamic fit cut offs and S12 for loadings). Internal reliability was good, Cronbach's $\alpha = .82$.

Fig. S1. Experiment 1: Three way interaction with headline type, headline congruence and past vote in the EU referendum for ratings of the extent to which headline is derogatory

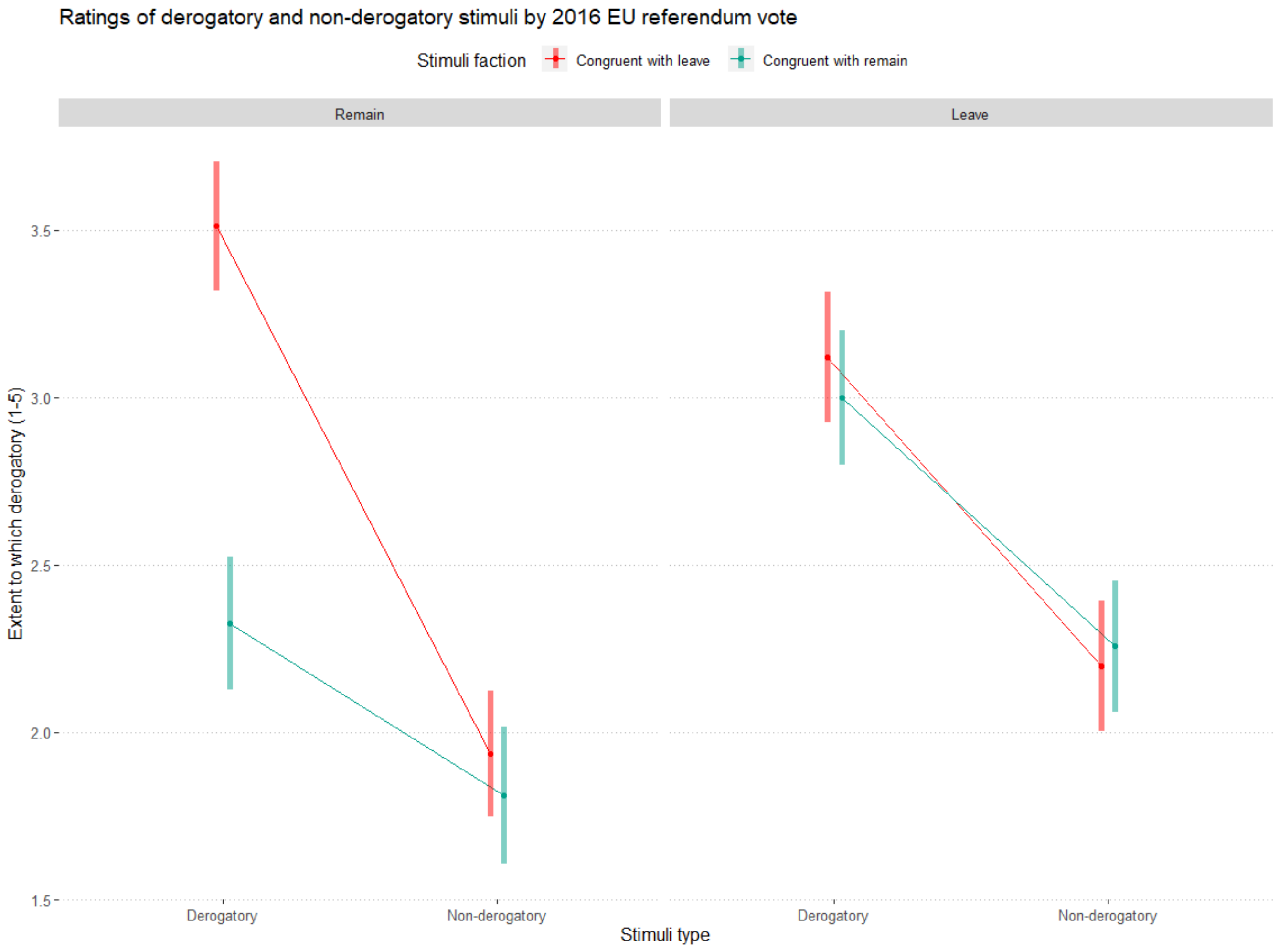

Table S1. Headline stimuli grouped by target group and theme for Experiment 1

	Stimuli type			
	Derogatory		Non-derogatory	
Stimuli headline theme	Remainer	Leaver	Remainer	Leaver
Failure to successfully execute Brexit	Bungled delivery: LEAVER incompetence extends BREXIT nightmare[1]	Remainer sabotage at heart of Brexit woes[1]	‘Unavoidable’: How Governments have struggled to avert Brexit related cost to economy	‘Missed opportunity’: How Governments have struggled to make the best of Brexit for economy
Media bias	‘It’s like some kind of evil plot’: A former insider’s take on the ‘desperate and pretty stupid’ BREXITEER media operation[2]	‘Make them look stupid’: A former insider’s take on the desperate evil REMAINER media plot to destroy Brexit[2]	Analysis reveals news Brexit bias: Majority of publications over-report benefits of Brexit	Analysis reveals news Brexit bias: Majority of publications under-report benefits of Brexit
Economic consequences	‘PUPPET’: ELITE BREXITEER economist attempts to downplay damage caused by BREXIT in CBI speech[3]	‘PUPPET’: ELITE REMAINER economist attempts to play down success of Brexit in CBI speech[3]	Economist counts the costs of Brexit to British economy in CBI speech	Economist lists off benefits of Brexit for British economy in CBI speech

[1] Employs scapegoating
[2] Employs emotional language
[3] Employs ad-hominem attacks

Table S2. Headline stimuli grouped by target group and theme for Experiment 2

	Stimuli type			
	**Derogatory**		**Non-derogatory**	
Stimuli headline theme	Remainer	Leaver	Remainer	Leaver
Failure to successfully execute Brexit	Bungled delivery: LEAVER incompetence extends BREXIT nightmare[1]	Remainer attack at heart of Brexit woes[1]	‘Unavoidable’: How Governments have struggled to avert Brexit related cost to economy	‘Missed opportunity’: How Governments have struggled to make the best of Brexit for economy
Media bias	‘It’s like some kind of evil plot’: A former insider’s take on the ‘desperate and pretty stupid’ BREXITEER media operation[2]	‘Make them look stupid’: A former insider’s take on the desperate evil REMAINER media plot to destroy Brexit[2]	Analysis reveals news Brexit bias: Majority of publications over-report benefits of Brexit	Analysis reveals news Brexit bias: Majority of publications under-report benefits of Brexit
Economic consequences	‘LIAR’: ELITE BREXITEER economist attempts to downplay damage caused by BREXIT in CBI speech[3]	‘LIAR’: ELITE REMAINER economist attempts to play down success of Brexit in CBI speech[3]	Economist counts the costs of Brexit to British economy in CBI speech	Economist lists off benefits of Brexit for British economy in CBI speech

[1] Employs scapegoating
[2] Employs emotional language
[3] Employs ad-hominem attacks

Table S3. Headline stimuli for Experiment 3

Headline set	Stimuli 1	Stimuli 2	Stimuli 3
1	Roe v. Wade: The fightback Begins in rage, fear and solidarity	This Supreme Court Roe v. Wade ruling is shockingly evil—and the court just made a huge mess for itself	Pelosi, blasting Row v. Wade decision, suggests Kavanaugh and Gorsuch lied at hearings
2	Voting Harder Won't Bring Back Roe	Coles Progressives rally after overturning of Roe v. Wade	The End of Roe Is Just the Beginning
3	Roe v. Wade based on a lie	Finally: The Death of Roe v. Wade	Victory of Truth: Joy and tears in Louisville the day after Roe v. Wade is struck down
4	Is There a 'Right to Privacy' Amendment?	Roe v. Wade ruling: CNN host criticizes Navarro for citing special needs relatives to justify Roe v. Wade	Supreme Court Justice Clarence Thomas says more rulings should be reconsidered after Roe is overturned

Table S4. Sample characteristics for Experiment 1

		Condition	
	Total (N=760)	Inoculation (N=363)	Control (N=397)
**Vote in 2016 EU referendum**			
I voted to Remain	378	180	198
	49.7%	49.6%	49.9%
I voted to Leave	285	138	147
	37.5%	38.0%	37.0%
I did not vote	91	42	49
	12.0%	11.6%	12.3%
Can't remember	6	3	3
	0.8%	0.8%	0.8%
**Gender**			
Man	359	170	189
	47.2%	46.8%	47.6%
Woman	401	193	208
	52.8%	53.2%	52.4%
**Highest level of education**			
None	27	16	11
	3.6%	4.4%	2.8%
Other	16	8	8
	2.1%	2.2%	2.0%
GCSE or equiv	102	57	45
	13.4%	15.7%	11.3%
A Level or equiv	159	69	90
	20.9%	19.0%	22.7%
Higher below degree	59	25	34
	7.8%	6.9%	8.6%
Degree	299	143	156
	39.3%	39.4%	39.3%
Don't know	14	5	9
	1.8%	1.4%	2.3%
Other technical things	84	40	44
	11.1%	11.0%	11.1%
**Age**			
18-24	69	35	34
	9.1%	9.6%	8.6%
25-49	307	145	162
	40.4%	39.9%	40.8%
50-64	241	115	126
	31.7%	31.7%	31.7%
65+	143	68	75
	18.8%	18.7%	18.9%

Table S5. Experiment 1: ANOVA 2 x 2 x 2 model with derogatory rating as the criterion and stimuli type, pastvote in the 2016 EU referendum and group stimuli is derogatory towards (outgroup) as factors

Effect	df	Mean Square	F	η^2_G	p
Stimuli type	1, 1237	1.56	176.36	.125	$< .001$
EU ref vote	1, 1237	1.56	12.31	.010	$< .001$
Outgroup	1, 1237	1.56	23.58	.019	$< .001$
Stimuli type : EU ref	1, 1237	1.56	2.25	.002	.133
Stimuli type : Outgroup	1, 1237	1.56	19.27	.015	$< .001$
EU ref vote : brexside	1, 1237	1.56	19.52	.016	$< .001$
Type : EU ref vote : Outgroup	1, 1237	1.56	9.72	.008	.002

Table S6. Regression coefficients for effect of video condition and stimuli type upon emoji reactions for Experiment 1

	Dependent variable:						
	Reacted	Angry reaction	Love reaction	Care reaction	Like reaction	Haha reaction	Sad reaction
Predictor	(1)	(2)	(3)	(4)	(5)	(6)	(7)
Video condition	-0.459^{***}	-0.355^{*}	-0.626	-16.421	0.000	0.276	-0.290
	(-0.766,-0.153)	(-0.717,0.007)	(-2.020,0.768)	(-3,219.283,3,186.440)	(-54,172.090,54,172.090)	(-0.338,0.890)	(-0.734,0.153)
Stimuli type	0.063	-0.058	-0.112	1.184	0.000	-0.758*	0.314
	(-0.246,0.372)	(-0.404,0.288)	(-1.309,1.085)	(-0.424,2.791)	(-54,128.300,54,128.300)	(-1.560,0.044)	(-0.085,0.712)
Video condition * Stimuli type	0.018	-0.236	1.360	15.850	-0.000	-0.142	0.102
	(-0.427,0.463)	(-0.780,0.307)	(-0.419,3.139)	(-3,187.012,3,218.712)	(-78,612.770,78,612.770)	(-1.263,0.979)	(-0.503,0.707)
Constant	0.322^{***}	-1.012^{***}	-4.034^{***}	-5.145^{***}	-26.566	-2.788***	-1.684***
	(0.108,0.535)	(-1.250,-0.773)	(-4.841,-3.227)	(-6.535,-3.755)	(-37,605.100,37,551.960)	(-3.240,-2.337)	(-1.975,-1.394)
Observations	1,268	1,268	1,268	1,268	1,268	1,268	1,268
Log Likelihood	-867.806	-667.771	-112.881	-58.731	-0.000	-242.506	-560.245
Nagelkerke R^2	.02	.02	.02	.07	.00	.02	.01

Unstandardized logistic regression coefficients (B) with 95% confidence intervals in parentheses.
$^{*}p < 0.10$; $^{**}p < 0.05$; $^{***}p < 0.01$

Table S7. Experiment 1: 2 x 2 between-within ANOVA model with sharing likelihood as the dependent variable and video condition and headline type (stimuli) as factors

Effect	Sum of Squares	df	Mean Square	F	η^2_G	p
Video condition	12.5	1, 648	1.28	9.76	0.013	.002
Stimuli	6	1, 648	0.23	26.56	0.006	.001
Video condition : Stimuli	0.00	1, 648	0.23	0.18	.001	.672

ANOVA with type III sums of squares

Table S8. Experiment 1: 2 x 2 between-within ANOVAs model with clicking likelihood as the dependent variable and video condition and headline type (stimuli) as factors

Effect	Sum of Squares	df	Mean Square	F	η^2_G	p
Video condition	6.9	1, 673	1.61	4.31	0.005	.038
Stimuli	24.4	1, 673	0.39	62.51	0.018	.001
Video condition : Stimuli	1.1	1, 673	0.39	2.78	.001	.096

ANOVA with type III sums of squares

Table S9. Experiment 1: 2 x 3 between-within ANOVA model with sharing likelihood on derogatory stimuli trials as the dependent variable and video condition and derogating technique employed as factors

Effect	df	Mean Square	F	η^2_G	p
Video condition	1, 682	0.73	10.29	0.015	.001
Technique	2, 682	0.73	0.26	< .001	.77
Video condition : Technique	2, 682	0.73	1.25	.004	.287

ANOVA with type III sums of squares

Table S10. Sample characteristics for Experiment 2

	Total (N=864)	Condition	
		Inoculation (N=436)	Control (N=428)
**Vote in 2016 EU referendum**			
I voted to Remain	410<br>47.5%	215<br>49.3%	195<br>45.6%
I voted to Leave	345<br>39.9%	179<br>41.1%	166<br>38.8%
I did not vote	102<br>11.8%	42<br>9.6%	60<br>14.0%
Can't remember	7<br>0.8%	0<br>0.0%	7<br>1.6%
**Gender**			
Male	429<br>49.7%	215<br>50.2%	214<br>49.1%
Female	435<br>50.3%	213<br>49.8%	222<br>50.9%
**Highest level of education**			
None	29<br>3.4%	17<br>3.9%	12<br>2.8%
Other	29<br>3.4%	13<br>3.0%	16<br>3.7%
GCSE or equiv	112<br>13.0%	55<br>12.6%	57<br>13.3%
A Level or equiv	167<br>19.3%	87<br>20.0%	80<br>18.7%
Higher below degree	74<br>8.6%	41<br>9.4%	33<br>7.7%
Degree	343<br>39.7%	170<br>39.0%	173<br>40.4%
DK	14<br>1.6%	5<br>1.1%	9<br>2.1%
Other technical things	96<br>11.1%	48<br>11.0%	48<br>11.2%
**Age**			
18-24	63<br>7.3%	26<br>6.0%	37<br>8.6%
25-49	343<br>39.8%	179<br>41.3%	164<br>38.3%
50-64	261<br>30.3%	126<br>29.1%	135<br>31.5%
65+	194<br>22.5%	102<br>23.6%	92<br>21.5%

Table S11. Model fit dynamic cut off and observed value for confirmatory factor analysis of Brexit identity strength subscales

Measure of fit	Observed value	Dynamic cut off
CFI	0.987	>.978
RMSEA	0.066	<.089
SRMR	0.025	<.029

Note: CFI = Comparative fit index; RMSEA = Root mean square error of approximation; SRMR = Standardized root square mean residual

Table S12. Unstandardized and standardized loadings of subscales onto factor 1 “Brexit identity strength”

Subscale item: Description	Unstandardized (SE)	Standardized
Identity strength 1: “We” instead of “they”	1 (-)	0.676
Identity strength 2: Personal insult	.851 (.065)	0.564
Identity strength 3: In common	.836(.056)	0.664
Identity strength 4: Connected	1.023 (.058)	0.83
Identity strength 5: Praise	.988 (.059)	0.755

*Note:*Dashes (-) indicate that the standard error was not estimated

Table S13. Sample characteristics for Experiment 3

	Total (N=1652)	Condition	
		Inoculation (N=840)	Control (N=812)
**Race**			
Asian	52 (3.1%)	30 (3.6%)	22 (2.7%)
Black	197 (11.9%)	104 (12.4%)	93 (11.5%)
Hispanic	230 (13.9%)	111 (13.2%)	119 (14.7%)
Middle Eastern	13 (0.8%)	5 (0.6%)	8 (1.0%)
Native American	13 (0.8%)	6 (0.7%)	7 (0.9%)
Other	27 (1.6%)	17 (2.0%)	10 (1.2%)
Two or more races	32 (1.9%)	15 (1.8%)	17 (2.1%)
White	1088 (65.9%)	552 (65.7%)	536 (66.0%)
**Presidential vote 2020**			
Joe Biden	834 (50.5%)	422 (50.2%)	412 (50.7%)
Donald Trump	545 (33.0%)	270 (32.1%)	275 (33.9%)
Did not vote for President	243 (14.7%)	132 (15.7%)	111 (13.7%)
Other	30 (1.8%)	16 (1.9%)	14 (1.7%)
**Gender**			
Female	879 (53.2%)	466 (55.5%)	413 (50.9%)
Male	773 (46.8%)	374 (44.5%)	399 (49.1%)
**Age group**			
18-24	134 (8.1%)	78 (9.3%)	56 (6.9%)
25-49	584 (35.4%)	301 (35.8%)	283 (34.9%)
50-64	497 (30.1%)	250 (29.8%)	247 (30.4%)
65+	437 (26.5%)	211 (25.1%)	226 (27.8%)
**Education level**			
4-year	337 (20.4%)	179 (21.3%)	158 (19.5%)
High school graduate	517 (31.3%)	258 (30.7%)	259 (31.9%)
Post-grad	198 (12.0%)	109 (13.0%)	89 (11.0%)
Some college	346 (20.9%)	175 (20.8%)	171 (21.1%)
2-year	182 (11.0%)	85 (10.1%)	97 (11.9%)
No HS	72 (4.4%)	34 (4.0%)	38 (4.7%)

Table S14. Experiment 2: Descriptive statistics for key measures total and as a function of video condition

				Video condition			
	Total			Control Video		Inoculation video	
Measure	Valid N	Mean	Std. Dev.	Mean	Std. Dev.	Mean	Std. Dev.
Polarisation score (0-100)	706	63.69	24.47	69.45	24.3	57.73	23.21
Sharing likelihood (1-4):							
Derogatory stimuli trials	688	1.61	0.86	1.71	0.91	1.5	0.79
Non-derogatory stimuli trials	675	1.76	0.88	1.85	0.93	1.66	0.81
Clicking likelihood (1-4):							
Derogatory stimuli trials	705	2.17	1.01	2.27	1.04	2.10	0.97
Non-derogatory stimuli trials	704	2.45	0.98	2.50	0.97	2.40	0.97
Brexit identity strength score (1-5)*	695	3.40	0.71	3.38	0.76	3.42	0.65

‘Valid N’ refers to the number of cases that gave a valid response i.e. those who did not answer ‘not sure’ or ‘don’t know’
*Asked before the video intervention

Table S15. Experiment 2: 2 x 2 between-within ANOVA model with sharing likelihood as the criterion and video condition and headline type (stimuli) as factors

Effect	Sum of Squares	df	Mean Square	F	η^2_G	p
Video condition	166.9	1, 860	12.67	13.18	0.013	< .001
Stimuli	115.3	1, 860	2.65	43.42	0.009	< .001
Video condition : Stimuli	0.6	1, 860	2.65	0.24	< .001	.627

ANOVA with type III sums of squares

Table S16. Experiment 2: 2 x 2 between-within model with clicking likelihood as the dependent variable and video condition and headline type (stimuli) as factors

Effect	Sum of Squares	df	Mean Square	F	η^2_G	p
Video condition	32	1, 860	15.22	2.12	.002	.146
Stimuli	176	1, 860	3.14	56.10	.011	$<$ .001
Video condition: Stimuli	3	1, 860	3.14	0.96	$<$ .001	.327

ANOVA with type III sums of squares

Table S17. Descriptive statistics for key measures total and as a function of video condition

				Video condition			
	Total			Control Video		Inoculation video	
Measure	Valid N	Mean	Std. Dev.	Mean	Std. Dev.	Mean	Std. Dev.
Polarisation score (0-100)	827	63.26	25.22	68.15	24.57	58.49	24.97
Sharing likelihood (1-10):							
Derogatory stimuli trials	862	3.45	2.79	3.78	2.96	3.12	2.58
Non-derogatory stimuli trials	862	3.96	2.78	4.26	2.91	3.67	2.61
Clicking likelihood (1-10):							
Derogatory stimuli trials	862	4.82	3.04	5.00	3.09	4.64	2.99
Non-derogatory stimuli trials	862	5.46	3.02	5.55	3.05	5.36	2.99
Brexit identity strength score (1-5)*	864	3.28	0.97	3.26	1.03	3.29	0.92

‘Valid N’ refers to the number of cases that gave a valid response i.e. those who did not answer ‘not sure’ or ‘don’t know’
*Asked before the video intervention

Table S18. Regression coefficients of the linear mixed model for Experiment 1

	M0			M1			M2			M3		
	Estimates	*CI*	*p*	*Estimates*	*CI*	*p*	*Estimates*	*CI*	*p*	*Estimates*	*CI*	*p*
(Intercept)	2.02	1.96 – 2.08	**<0.001**	2.10	2.03 – 2.18	**<0.001**	2.00	1.92 – 2.08	**<0.001**	2.02	1.93 – 2.10	**<0.001**
cond [Experimental]				-0.18	-0.29 – -0.07	**0.002**	-0.18	-0.29 – -0.07	**0.002**	-0.22	-0.34 – -0.09	**0.001**
derogatory [Issue]							0.21	0.16 – 0.26	**<0.001**	0.18	0.11 – 0.25	**<0.001**
cond [Experimental] × derogatory [Issue]										0.07	-0.03 – 0.17	0.185
**Random Effects**												
2	0.49			0.49			0.47			0.47		
00	0.50 ID			0.50 ID			0.50 ID			0.50 ID		
ICC	0.51			0.50			0.51			0.51		
N	750 ID			750 ID			750 ID			750 ID		
Observations	2772			2772			2772			2772		
BF Inclusion	NA			2.14			9.94e+11			0.046		

Table S19. Regression coefficients of the linear mixed model for Experiment 2

	M0			M1			M2			M3		
	Estimates	*CI*	*p*	*Estimates*	*CI*	*p*	*Estimates*	*CI*	*p*	*Estimates*	*CI*	*p*
(Intercept)	4.42	4.26 – 4.58	**<0.001**	4.65	4.42 – 4.88	**<0.001**	4.50	4.26 – 4.74	**<0.001**	4.51	4.27 – 4.76	**<0.001**
cond [Experimental]				-0.45	-0.77 – -0.12	**0.007**	-0.45	-0.77 – -0.13	**0.007**	-0.48	-0.83 – -0.13	**0.007**
derogatory [Issue]							0.33	0.20 – 0.47	**<0.001**	0.30	0.10 – 0.50	**0.003**
cond [Experimental] × derogatory [Issue]										0.07	-0.21 – 0.35	0.617
**Random Effects**												
2	4.19			4.19			4.15			4.15		
00	4.88 ID			4.83 ID			4.86 ID			4.86 ID		
ICC	0.54			0.54			0.54			0.54		
N	862 ID			862 ID			862 ID			862 ID		
Observations	3448			3448			3448			3448		
BF Inclusion	NA			0.666			1.09e+03			0.019		

Table S20. Regression coefficients of the linear mixed model for Experiment 3

	M0			M1			M2			M3		
	Estimates	*CI*	*p*	*Estimates*	*CI*	*p*	*Estimates*	*CI*	*p*	*Estimates*	*CI*	*p*
(Intercept)	2.90	2.86 – 2.94	**<0.001**	3.07	3.01 – 3.13	**<0.001**	3.10	3.04 – 3.17	**<0.001**	3.15	3.08 – 3.21	**<0.001**
cond [Experimental]				-0.33	-0.42 – -0.25	**<0.001**	-0.33	-0.42 – -0.25	**<0.001**	-0.42	-0.52 – -0.33	**<0.001**
sharing [issue]							-0.06	-0.10 – -0.02	**0.002**	-0.15	-0.21 – -0.10	**<0.001**
cond [Experimental] × derogatory [Issue]										0.18	0.11 – 0.26	**<0.001**
**Random Effects**												
2	0.92			0.92			0.92			0.92		
00	0.69 participant_id			0.66 participant_id			0.66 participant_id			0.67 participant_id		
ICC	0.43			0.42			0.42			0.42		
N	1652 participant_id			1652 participant_id			1652 participant_id			1652 participant_id		
Observations	9909			9909			9909			9909		
BF Inclusion	NA			1.18e+10			1.45			609.92		

Supplementary References

1. H Singmann, B Bolker, J Westfall, F Aust, MS Ben-Shachar, *afex: Analysis of Factorial Experiments*, (2023) R package version 1.3-0.
2. R Core Team, *R: A Language and Environment for Statistical Computing* (R Foundation for Statistical Computing, Vienna, Austria), (2022).
3. D Tingley, T Yamamoto, K Hirose, L Keele, K Imai, mediation: R package for causal mediation analysis. *J. Stat. Softw.* **59**, 1–38 (2014).
4. J Fox, S Weisberg, *An R Companion to Applied Regression.* (Sage, Thousand Oaks CA), Third edition, (2019).
5. D Lüdecke, MS Ben-Shachar, I Patil, P Waggoner, D Makowski, performance: An R package for assessment, comparison and testing of statistical models. *J. Open Source Softw.* **6**, 3139 (2021).
6. RM Baron, DA Kenny, The moderator-mediator variable distinction in social psychological research: Conceptual, strategic, and statistical considerations. *J. Pers. Soc. Psychol.* **51**, 1173–1182 (1986).
7. S Hobolt, J Tilley, *British Politics after Brexit.* p. 39–42 (2022).
8. HF Kaiser, WB Michael, Domain validity and generalizability. *Educ. Psychol. Meas.* **35**, 31–35 (1975).
9. MS Bartlett, A further note on tests of significance in factor analysis. *Br. J. Stat. Psychol.* **4**, 1–2 (1951).
10. RJ Vandenberg, Introduction: Statistical and methodological myths and urban legends. *Organ. Res. Methods* **9**, 194–201 (2006).
11. D McNeish, MG Wolf, Dynamic fit index cutoffs for confirmatory factor analysis models. *Psychol. Methods* **28** (2021).
