# Supplementary material for: Inoculation reduces social media engagement with affectively polarized content in the UK and US
Source: Commun Psychol. 2025 Jan 26;3:11. doi: 10.1038/s44271-025-00189-7 (PMC11769841; doi:10.1038/s44271-025-00189-7)
Supplement: Supplementary file 3 — Reporting Summary [file 44271_2025_189_MOESM3_ESM.pdf]

nature portfolio

Corresponding author(s): Almog Simchon

Last updated by author(s): Dec 19, 2024

Reporting Summary

Nature Portfolio wishes to improve the reproducibility of the work that we publish. This form provides structure for consistency and transparency in reporting. For further information on Nature Portfolio policies, see our Editorial Policies and the Editorial Policy Checklist.

Statistics

For all statistical analyses, confirm that the following items are present in the figure legend, table legend, main text, or Methods section.

n/a	Confirmed	
☐	☒	The exact sample size (*n*) for each experimental group/condition, given as a discrete number and unit of measurement
☐	☒	A statement on whether measurements were taken from distinct samples or whether the same sample was measured repeatedly
☐	☒	The statistical test(s) used AND whether they are one- or two-sided<br>*Only common tests should be described solely by name; describe more complex techniques in the Methods section.*
☐	☒	A description of all covariates tested
☐	☒	A description of any assumptions or corrections, such as tests of normality and adjustment for multiple comparisons
☐	☒	A full description of the statistical parameters including central tendency (e.g. means) or other basic estimates (e.g. regression coefficient) AND variation (e.g. standard deviation) or associated estimates of uncertainty (e.g. confidence intervals)
☐	☒	For null hypothesis testing, the test statistic (e.g. F, t, r) with confidence intervals, effect sizes, degrees of freedom and P value noted<br>*Give P values as exact values whenever suitable.*
☐	☒	For Bayesian analysis, information on the choice of priors and Markov chain Monte Carlo settings
☐	☒	For hierarchical and complex designs, identification of the appropriate level for tests and full reporting of outcomes
☐	☒	Estimates of effect sizes (e.g. Cohen's *d*, Pearson's *r*), indicating how they were calculated

Our web collection on statistics for biologists contains articles on many of the points above.

Software and code

Policy information about availability of computer code

Data collection	YouGov
Data analysis	R, RStudio, tidyverse, emmeans

For manuscripts utilizing custom algorithms or software that are central to the research but not yet described in published literature, software must be made available to editors and reviewers. We strongly encourage code deposition in a community repository (e.g. GitHub). See the Nature Portfolio guidelines for submitting code & software for further information.

Data

Policy information about availability of data

All manuscripts must include a data availability statement. This statement should provide the following information, where applicable:

- Accession codes, unique identifiers, or web links for publicly available datasets
- A description of any restrictions on data availability
- For clinical datasets or third party data, please ensure that the statement adheres to our policy

All data used in this article are publicly available at https://zenodo.org/record/8343729

Human research participants

Policy information about studies involving human research participants and Sex and Gender in Research.

Reporting on sex and gender	Experiment 1: 359 men, 401 women<br>Experiment 2: 429 men, 435 women<br>Experiment 3: 773 men, 879 women<br>Experiment 4: 336 men, 353 women, 2 data unavailable<br>Experiment 5: 322 men, 368 women, 2 prefer not to say<br>Experiment 6: 312 men, 374 women, 3 prefer not to say
Population characteristics	Experiments 1 & 2: Participants were recruited via a third-party online panel provider (YouGov) in a politically representative sample of adults in Great Britain.<br>Experiment 3: A representative American sample via YouGov.<br>Experiment 4: A representative American sample via Prolific<br>Experiment 5: Prolific users based in the US who have been pre-screened for the views on abortion<br>Experiment 6: Prolific users based in the US who have been pre-screened for the views on abortion
Recruitment	YouGov, Prolific
Ethics oversight	University of Bristol's Psychology Ethics Committee, approval nos.: 10176 and 11063

Note that full information on the approval of the study protocol must also be provided in the manuscript.

Field-specific reporting

Please select the one below that is the best fit for your research. If you are not sure, read the appropriate sections before making your selection.

☐ Life sciences ☒ Behavioural & social sciences ☐ Ecological, evolutionary & environmental sciences

For a reference copy of the document with all sections, see nature.com/documents/nr-reporting-summary-flat.pdf

Behavioural & social sciences study design

All studies must disclose on these points even when the disclosure is negative.

Study description	Experimental quantitative study (2x2 between-within design) where participants are assigned into an experimental and control group, shown several political headlines and need to respond on their clicking and sharing likelihood
Research sample	Adults in the UK and US via YouGov and US adults via Prolific
Sampling strategy	Experiment 1: Participants were recruited via a third-party online panel provider (YouGov) in March 2022. The study was included on three waves of a regular omnibus survey that employs quota sampling to recruit a politically representative sample of 1,600 adults in Great Britain. Only participants who identified as a ``Leaver'' or a ``Remainer'' and reported having previously shared political content on social media (see measures) were included. Due to these stringent inclusion criteria, only around 300 participants per wave of data collection qualified. Our target sample size was achieved after the first two waves of data collection, however due to an imbalance between Leavers and Remainers in the sample, we required a third wave, that only targeted Leavers. The final analysis included 760 participants (309 Leavers and 451 Remainers)<br>Experiment 2: Participants were again recruited via YouGov, with the same inclusion criteria as in Experiment 1. To replicate the same primary analyses of Experiment 1, we again pursued a sample size of 508 Leaver and Remainer participants who reported sharing political content on social media. In total, we ran four waves of data collection that recruited a total of 864 respondents (378 Leavers and 486 Remainers), aged 18-86 for our final analysis<br>Experiment 3: Our target and pre-registered sample size was 1,540. However, as the data collection was part of the Polarization Research Lab's Request for Proposals, the actual sample size exceeded this number, totalling 2,000 participants recruited from a representative American sample via YouGov<br>Experiment 4: Participants were recruited via Prolific (US sample). Our pre-registered target sample size was 690 participants. Based on a power analysis, we aimed to collect 620 participants to achieve 80% power, assuming a hypothesized effect size of d=0.2 and an alpha level of 0.05. Anticipating that approximately 10% of the sample would be excluded, we adjusted our final target to 690 participants. Participants received £0.90 for their participation in the study.In line with our pre-registered analysis plan, we excluded participants who completed the writing task unusually quickly. Specifically, we applied the lenient exclusion criterion recommended by Greszki et al. (2015), which flags individuals whose completion time is 50% faster than the median for their respective group. This approach identifies those with abnormally fast response behaviors. Although we initially recruited 692 participants, applying this criterion reduced the final sample size to 599.Our sample was designed to be representative of the US population across both demographic variables and political affiliation. The political breakdown of the final sample was as follows: 200 Democrats, 184 Republicans, 210 Independents, and 5 participants who preferred not to disclose their affiliation.<br>Experiment 5: Participants were recruited via Prolific (US sample). Similar to Experiment 4, our pre-registered target sample size was

690 participants. In line with our pre-registered analysis plan, we excluded participants who completed the writing task unusually quickly. Due to a higher-than-expected exclusion rate in Experiment 4, we applied an even more lenient exclusion criterion, flagging individuals whose completion time was 50% faster than the 70th percentile based on time spent on the writing task. Additionally, we asked participants if they had used AI to complete the task, informing them that such use would affect their compensation (£0.90).We initially recruited 691 participants. Applying the speeding criterion resulted in the exclusion of 68 participants, while the AI criterion excluded an additional 22. The final sample size was 645 participants, with 326 identifying as pro-choice and 319 as pro-life (gathered from Prolific pre-screeners). The political breakdown of the sample was as follows: 223 Democrats, 233 Republicans, 177 Independents, and 12 participants who preferred not to disclose their affiliation.
Experiment 6: Participants were recruited via Prolific (US sample). As in Experiments 4 and 5, our pre-registered target sample size was 690 participants. We applied the same exclusion criteria as in Experiment 5 and compensated participants £0.90 for their participation.We initially recruited 689 participants. After applying the speeding criterion, 34 participants were excluded, and an additional 23 were excluded based on the AI usage criterion. This left a final sample size of 634 participants, with 322 identifying as pro-choice and 312 as pro-life (based on Prolific pre-screening). The political breakdown of the sample was as follows: 209 Democrats, 254 Republicans, 158 Independents, and 13 participants who preferred not to disclose their affiliation.

Data collection

Online via YouGov and Prolific

Timing

Experiment 1: March 2022
Experiment 2: November-December 2022
Experiment 3: November 2022
Experiment 4: August 5 2024
Experiment 5: August 15 2024
Experiment 6: September 12 2024

Data exclusions

Experiment 3: In accordance with our pre-registered analysis plan, we excluded Independents (N = 348)
Experiment 4: In accordance with our pre-registered analysis plan, participants who completed the writing task unusually quickly (N = 93)
Experiment 5: In accordance with our pre-registered analysis plan, participants who completed the writing task unusually quickly (N = 68), and have confirmed they used AI in their response (N = 22)
Experiment 6: In accordance with our pre-registered analysis plan, participants who completed the writing task unusually quickly (N = 34), and have confirmed they used AI in their response (N = 23)

Non-participation

NA

Randomization

Random assignment to groups

Reporting for specific materials, systems and methods

We require information from authors about some types of materials, experimental systems and methods used in many studies. Here, indicate whether each material, system or method listed is relevant to your study. If you are not sure if a list item applies to your research, read the appropriate section before selecting a response.

Materials & experimental systems

n/a	Involved in the study
☐	☐ Antibodies
☐	☐ Eukaryotic cell lines
☐	☐ Palaeontology and archaeology
☐	☐ Animals and other organisms
☐	☐ Clinical data
☐	☐ Dual use research of concern

Methods

n/a	Involved in the study
☐	☐ ChIP-seq
☐	☐ Flow cytometry
☐	☐ MRI-based neuroimaging

Antibodies

Antibodies used

Describe all antibodies used in the study; as applicable, provide supplier name, catalog number, clone name, and lot number.

Validation

Describe the validation of each primary antibody for the species and application, noting any validation statements on the manufacturer's website, relevant citations, antibody profiles in online databases, or data provided in the manuscript.

Eukaryotic cell lines

Policy information about cell lines and Sex and Gender in Research

Cell line source(s)

State the source of each cell line used and the sex of all primary cell lines and cells derived from human participants or vertebrate models.

Authentication

Describe the authentication procedures for each cell line used OR declare that none of the cell lines used were authenticated.

Mycoplasma contamination	*Confirm that all cell lines tested negative for mycoplasma contamination OR describe the results of the testing for mycoplasma contamination OR declare that the cell lines were not tested for mycoplasma contamination.*
Commonly misidentified lines (See ICLAC register)	*Name any commonly misidentified cell lines used in the study and provide a rationale for their use.*

Palaeontology and Archaeology

Specimen provenance	*Provide provenance information for specimens and describe permits that were obtained for the work (including the name of the issuing authority, the date of issue, and any identifying information). Permits should encompass collection and, where applicable, export.*
Specimen deposition	*Indicate where the specimens have been deposited to permit free access by other researchers.*
Dating methods	*If new dates are provided, describe how they were obtained (e.g. collection, storage, sample pretreatment and measurement), where they were obtained (i.e. lab name), the calibration program and the protocol for quality assurance OR state that no new dates are provided.*

☐ Tick this box to confirm that the raw and calibrated dates are available in the paper or in Supplementary Information.

Ethics oversight	*Identify the organization(s) that approved or provided guidance on the study protocol, OR state that no ethical approval or guidance was required and explain why not.*

Note that full information on the approval of the study protocol must also be provided in the manuscript.

Animals and other research organisms

Policy information about studies involving animals; ARRIVE guidelines recommended for reporting animal research, and Sex and Gender in Research

Laboratory animals	*For laboratory animals, report species, strain and age OR state that the study did not involve laboratory animals.*
Wild animals	*Provide details on animals observed in or captured in the field; report species and age where possible. Describe how animals were caught and transported and what happened to captive animals after the study (if killed, explain why and describe method; if released, say where and when) OR state that the study did not involve wild animals.*
Reporting on sex	*Indicate if findings apply to only one sex; describe whether sex was considered in study design, methods used for assigning sex. Provide data disaggregated for sex where this information has been collected in the source data as appropriate; provide overall numbers in this Reporting Summary. Please state if this information has not been collected. Report sex-based analyses where performed, justify reasons for lack of sex-based analysis.*
Field-collected samples	*For laboratory work with field-collected samples, describe all relevant parameters such as housing, maintenance, temperature, photoperiod and end-of-experiment protocol OR state that the study did not involve samples collected from the field.*
Ethics oversight	*Identify the organization(s) that approved or provided guidance on the study protocol, OR state that no ethical approval or guidance was required and explain why not.*

Note that full information on the approval of the study protocol must also be provided in the manuscript.

Clinical data

Policy information about clinical studies

All manuscripts should comply with the ICMJE guidelines for publication of clinical research and a completed CONSORT checklist must be included with all submissions.

Clinical trial registration	*Provide the trial registration number from ClinicalTrials.gov or an equivalent agency.*
Study protocol	*Note where the full trial protocol can be accessed OR if not available, explain why.*
Data collection	*Describe the settings and locales of data collection, noting the time periods of recruitment and data collection.*
Outcomes	*Describe how you pre-defined primary and secondary outcome measures and how you assessed these measures.*

Dual use research of concern

Policy information about dual use research of concern

Hazards

Could the accidental, deliberate or reckless misuse of agents or technologies generated in the work, or the application of information presented in the manuscript, pose a threat to:

No	Yes	
☐	☐	Public health
☐	☐	National security
☐	☐	Crops and/or livestock
☐	☐	Ecosystems
☐	☐	Any other significant area

Experiments of concern

Does the work involve any of these experiments of concern:

No	Yes	
☐	☐	Demonstrate how to render a vaccine ineffective
☐	☐	Confer resistance to therapeutically useful antibiotics or antiviral agents
☐	☐	Enhance the virulence of a pathogen or render a nonpathogen virulent
☐	☐	Increase transmissibility of a pathogen
☐	☐	Alter the host range of a pathogen
☐	☐	Enable evasion of diagnostic/detection modalities
☐	☐	Enable the weaponization of a biological agent or toxin
☐	☐	Any other potentially harmful combination of experiments and agents

ChIP-seq

Data deposition

☐ Confirm that both raw and final processed data have been deposited in a public database such as GEO.

☐ Confirm that you have deposited or provided access to graph files (e.g. BED files) for the called peaks.

Data access links<br>*May remain private before publication.*	*For "Initial submission" or "Revised version" documents, provide reviewer access links. For your "Final submission" document, provide a link to the deposited data.*
Files in database submission	*Provide a list of all files available in the database submission.*
Genome browser session<br>(e.g. UCSC)	*Provide a link to an anonymized genome browser session for "Initial submission" and "Revised version" documents only, to enable peer review. Write "no longer applicable" for "Final submission" documents.*

Methodology

Replicates	*Describe the experimental replicates, specifying number, type and replicate agreement.*
Sequencing depth	*Describe the sequencing depth for each experiment, providing the total number of reads, uniquely mapped reads, length of reads and whether they were paired- or single-end.*
Antibodies	*Describe the antibodies used for the ChIP-seq experiments; as applicable, provide supplier name, catalog number, clone name, and lot number.*
Peak calling parameters	*Specify the command line program and parameters used for read mapping and peak calling, including the ChIP, control and index files used.*
Data quality	*Describe the methods used to ensure data quality in full detail, including how many peaks are at FDR 5% and above 5-fold enrichment.*
Software	*Describe the software used to collect and analyze the ChIP-seq data. For custom code that has been deposited into a community repository, provide accession details.*

Flow Cytometry

Plots

Confirm that:

☐ The axis labels state the marker and fluorochrome used (e.g. CD4-FITC).

☐ The axis scales are clearly visible. Include numbers along axes only for bottom left plot of group (a 'group' is an analysis of identical markers).

☐ All plots are contour plots with outliers or pseudocolor plots.

☐ A numerical value for number of cells or percentage (with statistics) is provided.

Methodology

Sample preparation	*Describe the sample preparation, detailing the biological source of the cells and any tissue processing steps used.*
Instrument	*Identify the instrument used for data collection, specifying make and model number.*
Software	*Describe the software used to collect and analyze the flow cytometry data. For custom code that has been deposited into a community repository, provide accession details.*
Cell population abundance	*Describe the abundance of the relevant cell populations within post-sort fractions, providing details on the purity of the samples and how it was determined.*
Gating strategy	*Describe the gating strategy used for all relevant experiments, specifying the preliminary FSC/SSC gates of the starting cell population, indicating where boundaries between "positive" and "negative" staining cell populations are defined.*

☐ Tick this box to confirm that a figure exemplifying the gating strategy is provided in the Supplementary Information.

Magnetic resonance imaging

Experimental design

Design type	*Indicate task or resting state; event-related or block design.*
Design specifications	*Specify the number of blocks, trials or experimental units per session and/or subject, and specify the length of each trial or block (if trials are blocked) and interval between trials.*
Behavioral performance measures	*State number and/or type of variables recorded (e.g. correct button press, response time) and what statistics were used to establish that the subjects were performing the task as expected (e.g. mean, range, and/or standard deviation across subjects).*

Acquisition

Imaging type(s)	*Specify: functional, structural, diffusion, perfusion.*
Field strength	*Specify in Tesla*
Sequence & imaging parameters	*Specify the pulse sequence type (gradient echo, spin echo, etc.), imaging type (EPI, spiral, etc.), field of view, matrix size, slice thickness, orientation and TE/TR/flip angle.*
Area of acquisition	*State whether a whole brain scan was used OR define the area of acquisition, describing how the region was determined.*

Diffusion MRI ☐ Used ☐ Not used

Preprocessing

Preprocessing software	*Provide detail on software version and revision number and on specific parameters (model/functions, brain extraction, segmentation, smoothing kernel size, etc.).*
Normalization	*If data were normalized/standardized, describe the approach(es): specify linear or non-linear and define image types used for transformation OR indicate that data were not normalized and explain rationale for lack of normalization.*
Normalization template	*Describe the template used for normalization/transformation, specifying subject space or group standardized space (e.g. original Talairach, MNI305, ICBM152) OR indicate that the data were not normalized.*
Noise and artifact removal	*Describe your procedure(s) for artifact and structured noise removal, specifying motion parameters, tissue signals and physiological signals (heart rate, respiration).*

Volume censoring | *Define your software and/or method and criteria for volume censoring, and state the extent of such censoring.*

Statistical modeling & inference

Model type and settings | *Specify type (mass univariate, multivariate, RSA, predictive, etc.) and describe essential details of the model at the first and second levels (e.g. fixed, random or mixed effects; drift or auto-correlation).*

Effect(s) tested | *Define precise effect in terms of the task or stimulus conditions instead of psychological concepts and indicate whether ANOVA or factorial designs were used.*

Specify type of analysis: ☐ Whole brain ☐ ROI-based ☐ Both

Statistic type for inference
(See Eklund et al. 2016) | *Specify voxel-wise or cluster-wise and report all relevant parameters for cluster-wise methods.*

Correction | *Describe the type of correction and how it is obtained for multiple comparisons (e.g. FWE, FDR, permutation or Monte Carlo).*

Models & analysis

n/a	Involved in the study
☐	☐ Functional and/or effective connectivity
☐	☐ Graph analysis
☐	☐ Multivariate modeling or predictive analysis

Functional and/or effective connectivity | *Report the measures of dependence used and the model details (e.g. Pearson correlation, partial correlation, mutual information).*

Graph analysis | *Report the dependent variable and connectivity measure, specifying weighted graph or binarized graph, subject- or group-level, and the global and/or node summaries used (e.g. clustering coefficient, efficiency, etc.).*

Multivariate modeling and predictive analysis | *Specify independent variables, features extraction and dimension reduction, model, training and evaluation metrics.*
